# To strengthen self-confidence as a step in improving prehospital youth laymen basic life support

**DOI:** 10.1186/s12873-020-0304-8

**Published:** 2020-01-30

**Authors:** Anna Abelsson, Per Odestrand, Annette Nygårdh

**Affiliations:** Jönköping University, School of Health Sciences, PO Box 1026, 551 11 Jönköping, Sweden

**Keywords:** Adult basic life support, Cardio pulmonary resuscitation CPR, First aid, Intervention, Layman, Self-confidence, Simulation

## Abstract

**Background:**

A rapid emergency care intervention can prevent the cardiac arrest from resulting in death. In order for Cardio Pulmonary Resuscitation (CPR) to have any real significance for the survival of the patient, it requires an educational effort educating the large masses of people of whom the youth is an important part. The aim of this study was to investigate the effect of a two-hour education intervention for youth regarding their self-confidence in performing Adult Basic Life Support (BLS).

**Methods:**

A quantitative approach where data consist of a pre- and post-rating of seven statements by 50 participants during an intervention by means of BLS theoretical and practical education.

**Results:**

The two-hour training resulted in a significant improvement in the participants’ self-confidence in identifying a cardiac arrest (pre 51, post 90), to perform compressions (pre 65, post 91) and ventilations (pre 64, post 86) and use a defibrillator (pre 61, post 81). In addition, to have the self-confidence to be able to perform, and to actually perform, first aid to a person suffering from a traumatic event was significantly improved (pre 54, post 89).

**Conclusion:**

By providing youth with short education sessions in CPR, their self-confidence can be improved. This can lead to an increased will and ability to identify a cardiac arrest and to begin compressions and ventilations. This also includes having the confidence using a defibrillator. Short education sessions in first aid can also lead to increased self-confidence, resulting in young people considering themselves able to perform first aid to a person suffering from a traumatic event. This, in turn, results in young people perceiveing themselves as willing to commence an intervention during a traumatic event. In summary, when the youth believe in their own knowledge, they will dare to intervene.

## Background

Sudden cardiac arrest is one of the most common preventable causes of death in the industrialized world [1]. Even after 5 minutes, the body begins to endure irreversible damage from oxygen deficiency [2]. A rapid intervention can prevent a cardiac arrest resulting in death [3]. The probability of surviving a cardiac arrest depends, among other things, on whether Cardio Pulmonary Resuscitation (CPR) is started immediately and the quality of the CPR [4, 5]. If CPR is started immediately and given with good quality, the risk of hypoxic damage decreases which dramatically increases the chance of survival [6]. Age of the patient also affects the outcome. The younger the patient, the greater the chance of survival [7, 8]. For any real significance for survival at a cardiac arrest, a CPR educational effort is required to teach the large masses in a population. The training targeted at those who are most likely to be present at a cardiac arrest can be a way of increasing the survival rate of the population [3].

In Swedish middle school (13–15 years old), BLS which include First aid and CPR according to the European Resuscitation Council guidelines [9], is mandatory [10, 11]. For students in high school (16–18 years), BLS is not mandatory but is described as an important part of handling emergencies and is vital knowledge for students [11]. Getting hands-on CPR training with manikins eliminates the barrier to performing CPR that otherwise may be experienced [12]. Increased knowledge contributes to increased safety in daring to intervene in emergencies.

However, saving lives is not just about the knowledge to know *How*, it’s also about the *Will* to save lives [3, 11]. Teaching children and adolescent BLS is also about promoting attitudes and contributing to a society in which people help each other [1, 11]. Fear of making mistakes and hurting the victim may be common reasons why individuals do not perform BLS [13–15].

The aim of this study was to investigate the effect of a two-hour education intervention for youth regarding their self-confidence in performing Adult Basic Life Support.

## Methods

This study had a quantitative approach where data consist of a pre- and post-rating of seven statements during an intervention by means of theoretical and practical BLS education.

### Participants

The study consisted of 50 high school students, 30 females and 20 males. The participants’ age ranged from 16 to 20 years (mean 17). Time since last BLS education ranged from 6 months to 2 years (mean 1 year). None of the participants had performed First aid or CPR on a human. Inclusion criteria were participation in BLS education. All participants received oral information about the study and were asked to participate voluntarily. All asked participants accepted.

### Data collection and intervention

The study was carried out in three steps during 1 day. In the first step, the participants rated seven statements regarding their perception of their knowledge of adult BLS and their self-confidence in performing BLS. The statements were abstracted from the theoretical and practical education arrangement, and pilot tested on persons not included in the study. The answers were rated 0–100 on a Likert scale were 0 represented No and 100 represented Yes. The statements to capture the participants’ self-confidence were:

*I have the knowledge to identify a person suffering from a sudden cardiac arrest.*

*I would perform chest compression on a person in need of it.*

*I would perform mouth to mouth ventilation to a person in need of it.*

*I would risk causing harm to the person I perform CPR on.*

*I would use a defibrillator if I had access to one during a cardiac arrest.*

*I have the knowledge to give first aid to a person suffering from a traumatic event.*

*I would give first aid to a person suffering from a traumatic event.*

In the second step, the participants took part in a two-hour theoretical and practical education by a certified instructor. The theory included anatomy and pathophysiology. The practical part included hands-on training in CPR was performed on a Brayden Pro (Innosonian®) according to the European Resuscitation Council guidelines [9]. The second step also included first aid practice on each other and on an actor impersonating a person suffering from a traumatic event.

In the third and last step, the participants rated the same statements as in the first step regarding perceptions and attitudes regarding adult BLS. The ratings in both the first and the third step were anonymized and collected by a person outside of the research group.

### Data analysis

The descriptive and analytic analysis was conducted using IBM Statistical Package for the Social Sciences (SPSS) 24.0. Descriptive analysis (central tendency and distribution) were used to describe the data. Analytic statistics (paired t-test) were used to compare the pre-and post-assessment.

## Results

The result shows a significant improvement in all seven statements regarding the participants’ self-confidence performing adult BLS (Table 1). The statements that improved the most were; *I have the knowledge to give first aid to a person suffering from a traumatic event,* improving from 46 to 86 on a 100-point Likert scale. The statement; *I have the knowledge to identify a person suffering from a sudden cardiac arrest*, improved from 51 to 90 on the 100-point Likert scale. The statement; *I would risk causing harm to the person I do CPR on*, improved by decreasing from 53 to only 14 on the 100-point Likert scale were 0 represented No. The statement; *I would perform chest compression on a person in need of it,* ranked highest in the post-test, 91 on the 100-point Likert scale.

**Table 1:** Presentation of pre- and post- measurement of the seven statements regarding the participants’ self-confidence performing adult BLS on a 0–100-point Likert scale with the range 0–100 were 0 is No and 100 is Yes

Item	Pre Mean (Std)	Post Mean (Std)	Differences mean pre-post	Sig
I have the knowledge to identify a person suffering from a sudden cardiac arrest	51 (27)	90 (13)	39	.00
range	0–100	50–100		
I would perform chest compression on a person in need of it.	65 (29)	91 (16)	26	.00
range	0–100	25–100		
I would perform mouth to mouth ventilation to a person in need.	64 (33)	86 (19)	22	.00
range	2–100	28–100		
I would risk causing harm to the person I perform CPR on.	53 (29)	14 (12)	39	.00
range	0–100	0–50		
I would use a defibrillator if I had access to one during a cardiac arrest.	61 (33)	81 (26)	20	.00
range	0–100	0–100		
I have the knowledge to give first aid to a person suffering from a traumatic event.	46 (30)	86 (16)	40	.00
range	0–100	50–100		
I would give first aid to a person suffering from a traumatic event.	54 (31)	89 (15)	35	.00
range	0–100	38–100		

## Discussion

The results show that the youths’ self-confidence improved after a two-hour training session as described previously [16]. The motivation to perform BLS increases with the number of training sessions [12, 17–20]. This is because the skill level is associated with self-confidence [21]. When the youth believe in their own knowledge, they will dare to intervene [17, 22]. Younger people have less resistance to train and perform BLS [1, 13]. Repeated BLS training at school affects the confidence levels and attitudes positively when emergency situations arise.

But confidence in performing CPR decreases with the deterioration of skills [23]. Refresher training, which focuses on skills and self-confidence rather than knowledge, should be carried out every 3 months due to the deterioration of skills [21]. It is important to note that individuals with lower self-confidence instead may avoid doing what they are unsure of. They will also lose the knowledge faster compared to individuals with better self-confidence [24].

Another important aspect is that CPR education by a role model may further enhance self-confidence for youths [12]. When a role model emphasizes that successful CPR is easy to learn and to perform, the individual’s assessment of their own ability to perform CPR is improved. One reason why youths’ self-confidence increased during this short education may be that the learning was done in a simulated lived and emotional experience. It has previously been described as a positive learning experience for younger people [25].

Ignorance of what a cardiac arrest is maybe a reason for why CPR is not always performed [13]. The rating of the youths’ self-confidence has risen from 51 in the pre-test to 90 in the post-test on the 100-point Likert scale of the statement; *I have the knowledge to identify a person suffering from a sudden cardiac arrest.* The spread of the rating at the post-test on this statement was 50–100 versus the pre-test being 0–100 points. It is a clear improvement of the participants’ self-confidence regarding CPR. This result can be compared with adults (18–91 years, mean 57 years) where approximately half of the participants believed being able to identify a cardiac arrest [13]. Participants’ self-confidence in the post-test is higher compared to a study that has shown that younger adolescents (10 to 12 years old), after training, has a good ability to identify a cardiac arrest [26].

In the result, the self-confidence regarding the statement; *I would perform chest compression on a person in need of it,* increased from 65 to 91 in the post-test. The more individuals in society who can perform CPR, have the self-confidence and willingness to perform chest compressions, the more increased likelihood that someone will actually perform CPR. The statement; *I would perform mouth to mouth ventilation to a person in need of it*, was rated in the pre-test as 64 and increased in the post-test to 86. This increase is a small improvement of the youths’ self-confidence in performing mouth to mouth ventilation. In real life events, there is a possibility for a high psychological barrier of youth to initiate mouth-to-mouth ventilation [27] resulting in en poorly performed ventilation of the patient [28]. Schroeder et al. [29], therefore, argues that, due to the decreasing importance of mouth-to-mouth ventilation in lay resuscitation in favor of chest compressions, specially adapted CPR-algorithms for school children could be discussed [29]. So far, all students in Sweden are trained in both compression and ventilation according to the European Resuscitation Council guidelines [9–11].

It is approximately 25–50% of patients with a sudden cardiac arrest that suffers from ventricular fibrillation at the onset of advanced life support and therefore in need of Automated External Defibrillator (AED) [2, 29–31]. Research shows that youth can easily handle an AED [32]. The statement; *I would Use a defibrillator* was rated 61 in the pre-test and increased to 81 in the post-test on the 100-point Likert scale. The self-confidence to use an AED leads to a more effective CPR and can also create a more motivated youth to intervene in a future medical emergency.

On the statement; *I have the knowledge to do first aid to a person suffering from a traumatic event* the youths’ self-confidence increased from 46 in the pre-test to 86 in the post-test. The increase for the statement; *I would give first aid to a person suffering from a traumatic event* went from 54 in the pre-test to 89 in the post-test which both are an increase. Teaching young people BLS contributes to a society in which we care to help each other. Increased knowledge contributes to increased safety in daring to intervene in emergencies. Saving lives is not just about knowledge- it is also about wanting to help. The Swedish curriculum indicates that students should develop knowledge of how to act in emergencies [11]. Students learn to help others and get a sense of responsibility for other people [1].

### Limitations

The BLS training has reduced anxieties over making mistakes and enhanced the youths’ self-confides, therefore, increased the youths’ willingness to help others. A limitation is that there hasn’t been any focus on how long this enhanced self-confidence remains with the youth. Since this study included youth from different schools in one area, the extent to which motivation and social structure influenced the results remains unclear.

## Conclusion

By providing youth with short education sessions in CPR, their self-confidence can be improved. This can lead to an increased will and ability to identify a cardiac arrest and to begin compressions and ventilations. This also includes having the confidence using a defibrillator. Short education sessions in first aid can also lead to increased self-confidence, resulting in young people considering themselves able to perform first aid to a person suffering from a traumatic event. This, in turn, results in young people perceiveing themselves as willing to commence an intervention during a traumatic event. In summary, when the youth believe in their own knowledge, they will dare to intervene.

## Data Availability

All data generated or analyzed during this study are included in this published article.

## Abbreviations

AED: Automated external defibrillator
BLS: Basic life support
CPR: Cardiopulmonary resuscitation
SPSS: Statistical Package for the Social Sciences
